# Paediatric Antimicrobial Stewardship for Respiratory Infections in the Emergency Setting: A Systematic Review

**DOI:** 10.3390/antibiotics10111366

**Published:** 2021-11-08

**Authors:** Keshani Weragama, Poonam Mudgil, John Whitehall

**Affiliations:** Department of Paediatrics, School of Medicine, Western Sydney University, Campbelltown, NSW 2560, Australia; kweragama@gmail.com (K.W.); John.Whitehall@westernsydney.edu.au (J.W.)

**Keywords:** paediatric antibiotic stewardship, paediatric antibiotic resistance, antimicrobial stewardship programs, ASP, antimicrobial resistance, respiratory tract infections, paediatric emergency department

## Abstract

Antimicrobial resistance occurs due to the propensity of microbial pathogens to develop resistance to antibiotics over time. Antimicrobial stewardship programs (ASPs) have been developed in response to this growing crisis, to limit unnecessary antibiotic prescription through initiatives such as education-based seminars, prescribing guidelines, and rapid respiratory pathogen (RRP) testing. Paediatric patients who present to the emergency setting with respiratory symptoms are a particularly high-risk population susceptible to inappropriate antibiotic prescribing behaviours and are therefore an ideal cohort for focused ASPs. The purpose of this systematic review was to assess the efficacy and safety of ASPs in this clinical context. A systematic search of PubMed, Medline, EMBASE and the Cochrane Database of Systematic Reviews was conducted to review the current evidence. Thirteen studies were included in the review and these studies assessed a range of stewardship interventions and outcome measures. Overall, ASPs reduced the rates of antibiotic prescription, increased the prescription of narrow-spectrum antibiotics, and shortened the duration of antibiotic therapy. Multimodal interventions that were education-based and those that used RRP testing were found to be the most effective. Whilst we found strong evidence that ASPs are effective in reducing antibiotic prescribing, further studies are required to assess whether they translate to equivalent clinical outcomes.

## 1. Introduction

Antimicrobial resistance (AMR) continues to present a growing public health challenge in an era of widespread antibiotic availability [1]. As resistance continues to rise, there is a substantial threat to the medical benefits of antibiotics and increasing mortality associated with drug-resistant infections [2]. Moreover, multidrug resistant bacteria have evolved over the past century as a result of their genetic capacities to exploit resistance genes and utilize horizontal gene transmission to develop numerous mechanisms of antibiotic resistance [3]. Strains including MRSA and VRE are of particular concern as they are responsible for significant morbidity and mortality in hospital and long-term care facilities and have recently become a major community-acquired pathogen [3,4]. Further, emerging strains such as Campylobacter species and *Streptococcus pneumoniae* pose a moderate to high risk, especially to vulnerable populations including preterm infants, those that are immunocompromised and the elderly [2,5,6].

The paediatric population in particular has become recognized as a nidus for the propagation of AMR in recent times [7]. Inappropriate rates of antibiotic prescription have been observed across several healthcare settings, from primary to secondary care, with a particular focus being placed on primary care [8]. Several studies have shown promise with regards to the use of ASP interventions in the primary care setting [9,10,11]. Further, recent reviews exploring paediatric ASPs in both inpatient and outpatient settings have observed the potential of these interventions in reducing antimicrobial use, healthcare costs and AMR [12]. The level of evidence surrounding the effectiveness of ASPs in the paediatric emergency department (ED) remains incoherent and requires further investigation. Following trauma, infections are the most common ED presentation in the paediatric population [13]. Due to the rare but potentially life-threatening nature of serious bacterial infections, there is a clear pattern of overprescription of antibiotics in the paediatric setting [14]. This pattern can be attributed to a number of factors including parental pressure, medical liability, diagnostic uncertainty as well as fear of adverse complications or death [14]. Antibiotics should, however, be used sparingly where there is a sufficient clinical indication or where the risk of missing a significant bacterial infection may cause significant morbidity or mortality.

Acute respiratory tract infections (ARTIs) are among the most common in children and account for a significant proportion of the antibiotics prescribed in paediatric emergency departments [15]. Studies have attributed up to 80% of antibiotic prescription in the paediatric ED setting to ARTIs [16]. With approximately 75% of ARTIs stemming from viral infections such as respiratory syncytial virus, this highlights an area of antibiotic misuse that must be addressed [17]. In some countries, vast distribution of vaccines including pneumococcal conjugate and *Haemophilus influenzae* Type B have had a greater effect in reducing the incidence of bacterial infections that cause severe morbidity in comparison to antibiotics [14]. Further, recent literature has found that antibiotic prescription rates for ARTIs are most commonly dependent on hospital protocol and has brought to light the overprescription of second-line antibiotics as an initial prescription [16]. These findings highlight areas of antibiotic prescription can be addressed to ensure appropriate antibiotic prescription.

Antimicrobial stewardship programs (ASPs) were introduced in healthcare settings to guide antibiotic prescribing by encouraging the use of narrow-spectrum antibiotics, optimizing dosages, and shortening the duration of antibiotics prescribed [18]. The concept of antimicrobial stewardship has been explored since 1996 and continues to evolve with the emergence of new resistance pathogens and the introduction of new antibiotics. Early ASPs focused on antibiotic de-escalation from empirical to targeted therapy [19]. More recent programs have utilized a multimodal approach, including education on antimicrobial usage, development and implementation of evidence-based guidelines to optimize antibiotic prescription [20].

It is important to assess how effective these programs are at addressing these concerns in order to make the necessary adjustments and continue to implement these strategies to help control the global burden of AMR. There are no current systematic reviews addressing the use of ASPs in the paediatric emergency department, with regards to respiratory infections. Our review will address this topic to support improvements in the delivery and quality of future paediatric ASPs.

The purpose of this systematic review was to assess the efficacy of ASPs in addressing suboptimal antibiotic prescribing for respiratory infections in the paediatric emergency setting. The primary outcome of the study was to determine whether the implementation of ASPs in paediatric emergency settings translated to judicious antibiotic prescribing in the form of narrow spectrum antibiotics, shorter courses, and reduced dosing. The secondary outcome was to determine whether the implementation of ASPs resulted in comparable clinical outcomes when compared to usual prescribing practices.

## 2. Materials and Methods

A systematic review of the literature was performed to identify primary journal articles that assessed the efficacy and safety of ASPs in guiding appropriate antibiotic prescribing. The Preferred Reporting Items for Systematic Reviews and Meta-analyses (PRISMA) guidelines were followed when performing the review.

### 2.1. Focused Question

Are antimicrobial stewardship programs for acute respiratory presentations in the paediatric emergency setting effective in narrowing antibiotic spectrum, reducing dosage, and shortening duration of antibiotic prescription?

### 2.2. PICO Question

P (population): paediatric patients (aged 3 months–18 years old) presenting to the emergency department with respiratory symptoms; 

I (intervention): implementation of antibiotic stewardship programs; 

C (comparison): usual care;

O (outcome): changes in antibiotic prescribing behaviours whilst maintaining patient outcomes.

### 2.3. Search Strategy

The review was performed on 28 August 2021 and included PubMed, MEDLINE, EMBASE and the Cochrane Database of Systematic Reviews for publications from 1 August 2001 to 31 July 2021 to include studies from the last twenty years. Briefly, specific search terms included ‘antimicrobial stewardship’, ‘antimicrobial control’, ‘paediatric’, ‘respiratory tract infection’ and ‘emergency department’. The complete search strategy used for MEDLINE is shown in Appendix A, which was then adapted for the remaining databases. Studies that were included were primary journal articles, had full text availability and in English language.

### 2.4. Eligibility Criteria

Inclusion criteria: all primary research articles that included children aged between 1 month–18 years who presented to an emergency department due to respiratory infections. Studies that implemented an ASP as an intervention and reported outcomes pertaining to efficacy or safety were included.

Exclusion criteria: articles were excluded if they were case series, letters, notes, conference abstracts, policy statements or opinion articles. ASPs implemented in paediatric settings other than emergency departments were excluded. For studies that included both adult and paediatric patients and the paediatric data was not available, the corresponding author was contacted, and the data requested. Studies reporting on antiviral and antifungal drugs were also excluded [21,22].

### 2.5. Study Selection

The titles and abstracts of studies that were identified by the search were screened and studies that were deemed relevant were further assessed using the full texts and reviewed in lieu of the inclusion and exclusion criteria. Additionally, reference lists of eligible studies and of relevant systematic reviews were examined to identify further studies. The search and study selection was conducted independently by two authors (KW and PM) and disagreements were resolved by consensus.

### 2.6. Study Quality & Risk of Bias

The quality of included articles was assessed using the Integrated Quality Criteria for Systematic Review of Multiple Study Designs (ICROMS) tool [23]. Only studies that met the minimum score and mandatory criteria according to the ICROMS tool were included in the final analysis.

### 2.7. Data Extraction

Data was extracted by KW from all eligible studies using a standardized data collection form that was constructed a priori. Variables of interest included study design, location and setting, details of ASP-implemented prescribing practices and patient outcomes.

## 3. Results

### 3.1. Search Results

**Figure 1:**
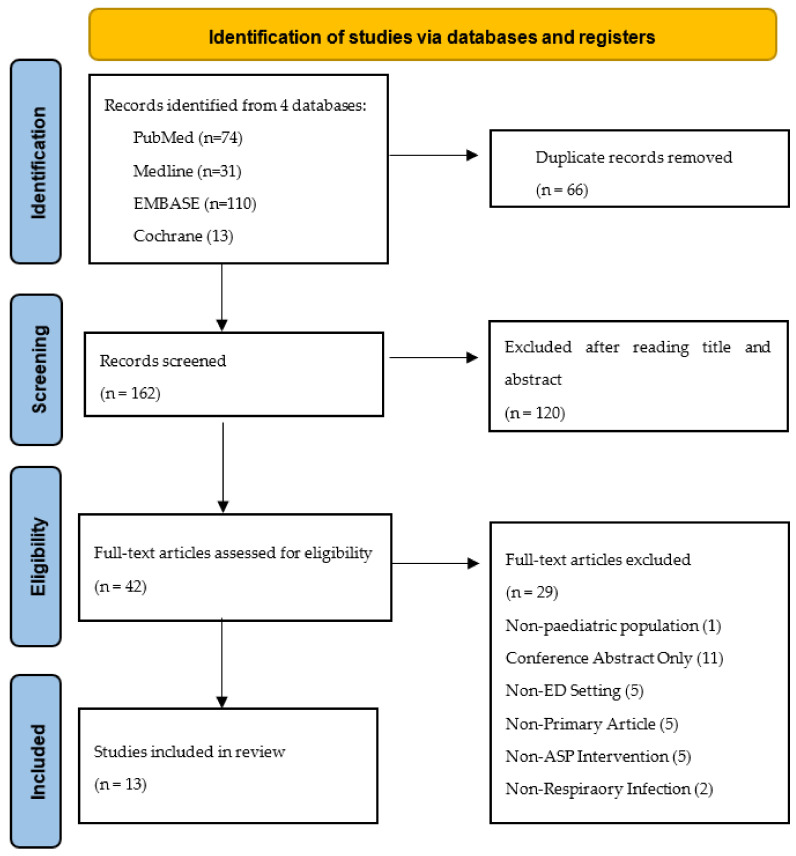
Preferred Reporting Items for Systematic Reviews and Meta-Analysis (PRISMA) flow diagram of study selection process.

In total, 228 articles were identified using the search strategy, with a filter limiting to titles published in English between 2001 and 2021. After removing 66 duplicates, the abstracts of the remaining 162 were screened and 120 were excluded for a variety of reasons including being a non-primary article, not having an ASP or QI intervention, not focusing on the paediatric population, not addressing respiratory tract presentations and for not being conducted in the emergency setting. Following the title and abstract screening, a total of 42 full-text articles were reviewed. Thirteen articles were eligible for final analysis (Figure 1).

### 3.2. Included Studies

A summary of the included studies is provided in Table 1. These studies were published between 2013 [24] and 2021 [25,26]. Four of the thirteen studies were randomized-control studies [25,27,28,29], three were cohort studies [24,30,31], one was a controlled interrupted time series analysis [13] and five were noncontrolled before-and-after studies [26,32,33,34,35]. The studies were located in a variety of countries and settings, with eight of thirteen studies being conducted in the United States [24,27,29,30,31,33,34,35], and the other studies being conducted in Taiwan, France, Canada, Japan and the Netherlands [13,25,26,28,32]. All studies were conducted within the emergency departments and urgent care of hospitals, however the number and type of hospitals varied between studies. Eight of thirteen studies reported single-centre interventions [24,26,27,30,31,32,33,35], whilst the other five conducted reported outcomes from multiple centres [13,25,28,29,34]. Further, six studies reported outcomes from paediatric hospitals only [24,25,26,31,35], one study used both paediatric and general hospitals [29], and the remaining five studies extracted paediatric data from a general hospital with adults and children [13,27,28,30,32]. The sample size was based on whether the article was reporting patient outcomes or physician outcomes but ranged from 26 [34] to 242,534 [13].

### 3.3. Intervention

The nature of ASP interventions varied greatly between included studies. Eight of these studies reported outcomes from education-based interventions which provided clinical decision tools and aids with current guidelines regarding appropriate antibiotic prescription [13,24,28,29,30,31,33,34]. The delivery of these interventions varied between studies but included team meetings and education seminars, distribution of physical copies of clinical decision tools, email notifications [13,24,28,29,30,31,33,34]. Further, intervention duration ranged from one month [33] to one year [13]. All eight studies used evidence-based interventions, with a majority of study interventions stemming from current clinical practice guidelines [13,24,28,30,33]. Rutman et al., developed a community-acquired pneumonia pathway during the study period based on the study population, current literature, and hospital guidelines [31]. Yadav et al., utilized interventions based on previous ASPs which were found to be effective in the outpatient setting [29]. Two of the education-based included studies also provided ongoing feedback to the physicians throughout the study as a further intervention [13,30]. Yadav et al., focused on feedback as an intervention by comparing two ASP interventions. Both interventions were based on the same campaign however the one intervention was enhanced with individualized auditing, feedback, peer comparisons and nudges [29]. Shishido et al., did not provide an education-based intervention but initiated a program that monitors the use of third generation cephalosporins (3GC) and published these prescribing patterns in monthly newsletters as a nudge-based ASP [26]. May et al., and Zhu et al., assessed the effectiveness of rapid respiratory panel (RRP) testing, to identify common viral and bacterial pathogens, as an intervention to reduce unnecessary antibiotic prescription [27,35]. Huang et al., conducted a retrospective study assessing the impact of a national pneumococcal conjugate vaccine (PCV13) between 2008–2012 and education-based nation-wide ASP conducted between 2013 and 2015 on antibiotic resistance to respiratory tract bacteria in children [32]. Pernica et al., observed the effectiveness of a short dose of amoxicillin in comparison to a long dose of amoxicillin to evaluate the stewardship concept of reduced length of antibiotic therapy [25].

### 3.4. Primary Outcome

The primary outcomes reported in the included studies are heterogeneous, ranging from antibiotic prescription rate [24] to susceptibility of respiratory pathogens to antimicrobial agents [32] (Table 2). Many studies reported the proportion of appropriate antibiotic prescriptions as a primary outcome, comparing rates both pre- and postintervention [13,24,27,28,29,30,34]. Two studies looked at the prescription rate of specific antibiotics, namely ampicillin, third generation cephalosporins [26,31]. McDaniel et al., explored whether physicians adhered to the diagnostic measures outlined in the clinical decision tool and then assessed therapeutic measures including changes in the prescription of macrolides and narrow spectrum antibiotics [33]. Zhu et al., focused on the number of days of antibiotic therapy and the number of patients who left ED with antibiotics [35]. Several studies evaluated clinical as a primary measure. Van de Maat et al.,, in addition to antibiotic prescription rate, also assessed strategy failure within a week of the initial ED visit [28]. Similarly, Forrest et al.,, in addition to appropriate use of antibiotics, also assessed physician and patient engagement and satisfaction with the intervention as a primary outcome [30]. Pernica et al., also explored clinical cure 14–21 days after presentation as a primary outcome [25]. Finally, Huang et al., retrospectively assessed the susceptibility of respiratory pathogens to antimicrobial agents over three major time periods [32].

### 3.5. Efficacy of ASPs

#### 3.5.1. Education-Based Interventions

Of the studies based on education-based interventions with clinical decision tools, a significant reduction of overall antibiotic use and inappropriate antibiotic prescription was reported by six studies [13,24,29,30,33,34]. Two studies found an increase in appropriate antibiotic prescription, however, did not reach statistical significance [28,31]. Two studies reported an increase in the use of narrow-spectrum antibiotics; McDaniel et al., observed a statistically significant increase of 8.3% (95% CI 21.5,15.2) and Rutman et al., observed a slight increase from 57 to 67% postintervention in the ED population [31,33].

#### 3.5.2. Feedback for STUDY Participants

Study interventions which provided ongoing feedback to participants throughout the study period showed a significant increase in appropriate antibiotic use. Ouldali et al., provided feedback during the first year of intervention and observed a −15.5%, *p* = 0.01 decrease in antibiotic prescription rate and with an estimated cumulative effect of intervention of −30.9% (95% CI −42.5, −20.1) [13]. It is however difficult to attribute this change to feedback only as Yadav et al., found no significant difference-in-differences between the arm of his study that received feedback in comparison to the group that did not receive feedback [29].

#### 3.5.3. RRP Testing

Of the two studies based on RRP testing interventions, both reported a decrease in antibiotic use [27,35]. However, only one paper reported a statistically significant reduction [27]. Although, Zhu et al., did not find a significant difference in the number of patients who received antibiotics, there was a statistically significant reduction (−32.3%, *p* < 0.001) in discharge antibiotic prescriptions between the number of patients testing positive with RRP in comparison to patients who were not tested [35].

#### 3.5.4. Impact of Vaccinations/Enhanced Antimicrobial Control

Huang et al., reported a significant reduction in the resistance of antibiotics in response to the pneumococcal vaccination program across Taiwan. Certain pathogens such as *H. influenzae* however, decreased in susceptibility throughout the study period. Further, the enhanced antibiotic strategy, including an education-based ASP towards the end of the study only showed a significant improvement of drug resistance in nosocomial pathogens and not community-associated pathogens [32].

### 3.6. Clinical Outcomes following ASP Search Results

Pernica et al., observed the effects of a short-course high-dose antibiotic (intervention) in comparison to a long course of high-dose antibiotics (control) on clinical cure between 14–21 days [25]. Clinical cure was clearly defined and included an initial improvement during the first 4 days, improvement in dyspnoea and work or breathing, only one or no fever spikes and a lack of further antibiotic prescription. Clinical cure at 14–21 days postintervention was observed in 85.7% in the intervention group and 84.1% in the control group (RD 0.023; 97.5% CI −0.061). Therefore, this study found that a short-course of antibiotics was comparable to a long-course in the treatment of community-acquired pneumonia presentations in the ED [25]. Van de Maat et al., also included strategy failure as a primary outcome following the utilization of a clinical decision tool. Strategy failure included a secondary antibiotic prescription, hospitalization, recurrent fever, oxygen dependency or further complications within 7 days. In this study, a significant decrease in strategy failure from 23% to 16% (aOR 0.53, 95% CI 0.32, 0.88, *p* = 0.01) was observed following intervention [28].

### 3.7. Risk of Bias

All included studies were assessed for bias using the ICROMS tool. All studies met the mandatory criteria and minimum score required to be deemed of fair quality and therefore were included for review (Appendix B, Appendix C and Appendix D)

## 4. Discussion

The global burden of AMR, particularly in the context of increased antibiotic use is rapidly increasing. ASPs were developed in response to this growing public health issue and are aimed at optimising antibiotic prescription, whilst maintaining patient care [36]. The emergency setting is imperative for efforts in antimicrobial stewardship due to the high frequency of infectious presentations and acute outpatient referrals [36]. Recent literature exploring the use of ASPs in the emergency setting have shown promise in promoting appropriate prescription of antibiotics [37,38,39]. Savoldi et al., associated a reduction in antibiotic costs and usage with the implementation of a general ED-based ASP [38]. Further, patient outcomes were also improved with an overall reduction in patient length of stay and reduced rates of *Clostridioides difficile* infection in patients admitted from ED [38]. May et al., assessed the effectiveness of a multifaceted ASP for ED patients with skin and soft tissue infections (SSTI) [39]. This study found only a modest improvement in appropriate antibiotic use, with a decrease in the duration of antibiotic therapies [39].

There is limited evidence surrounding the use of ASPs in the ED setting for the paediatric population, in particular with regard to ARTIs. 

This systematic review looking at the effectiveness of ASPs for respiratory presentations in the paediatric emergency setting shows that multiple methods of delivering education-based ASPs translate to improved antibiotic prescribing and equivalent or improved clinical outcomes and safety, which are sustainable. Further, the availability of diagnostic tools such as RRP testing facilities shows promise in increasing the rate of appropriate antibiotic prescribing.

When evaluating the efficacy of ASPs, the most commonly used measures included proportion of appropriate first-line antibiotic use and broad-spectrum antibiotics. The effects of overall antibiotic use were well reported by the included studies, and the postintervention difference was statistically significant in nine of thirteen studies [13,24,26,27,29,30,33,34,35]. As effects were seen in a variety of ASP multimodal intervention types, it can be difficult to ascertain which strategies optimised ASP delivery and therefore efficacy. A number of included studies implemented a comparable approach when delivering ASPs, with an education-based seminar followed by updates to physical handbooks and electronic medical record systems [13,24,28,29,30,31,33,34]. One of these studies by Ambroggio et al., implemented a ‘level of reliability’ (LOR) measure which indicates the likelihood of an intervention to fail in the system over time [24]. Based on their results, the study allocated their ASP delivery methods including guideline seminars, recommendations in medical staff updates, nurse flag cards, index cards with appropriate antibiotic information and resident reports as a level one LOR, indicating the intervention would only fail once or twice for every ten encounters [24]. This is in line with current literature which supports the use of multimodal, education-based ASPs, attributing these programs to changes in knowledge, attitude, and quality of antibiotic prescription [40,41,42]. On the other hand, a retrospective study by Huang et al., found that the pneumococcal vaccine significantly improved AMR in Taiwan, however, although the education-based ASP improved drug resistance of nosocomial pathogens, it did not have a significant effect on community-associated pathogens [32]. 

Further, two other included studies although implementing education-based clinical tools, reported a decrease in antibiotic use, however, these results did not reach statistical significance [28,31]. Rutman et al., attributed this to contextual factors including leadership support and hospital culture, whereas Van de Maat et al., recognised this lack of significance was likely due to a low number of baseline prescription rates in comparison to other studies and a large proportion of high-risk patients present during the study period [28,31]. Therefore, further research with sufficient power and adequate control of confounding factors should be conducted to confirm these findings. 

There is conflicting evidence regarding the use of feedback techniques when delivering an ASP, with some studies reporting a significant change in the prescription of targeted antibiotics [43], and others not seeing an improvement [44]. Two included studies provided physician feedback as part of their ASP intervention, and both found a statistically significant reduction in rates of antibiotic prescription [13,29]. In the RCT conducted by Yadav et al., feedback was only provided for the intervention arm of the study, and a statistically significant reduction in inappropriate prescribing from 2.2% (95% CI 1.0–3.4) to 1.5% (95%CI 0.7–2.3) [OR 0.67] was reported. This study, however, included both adult and paediatric populations in this analysis, therefore future RCTs need to be conducted to confirm this results in the paediatric population alone [29].

In the adult population, a significant reduction in inappropriate antibiotic prescription (41.6% versus 11%, *p* < 0.0001) after the utilisation of RRP has been reported [45]. This is in line with recent literature highlighting the potential of rapid respiratory testing to increase the appropriate prescribing of antimicrobial agents [46]. This evidence, however, is limited for the paediatric population. May et al., conducted an RCT assessing the effectiveness of RRP testing in comparison to the usual care on antimicrobial stewardship [27]. The study was ceased before it was able to reach the required sample size, however displayed an overall reduction of antibiotic use with RRP testing (−12% difference; *p* = 0.06) that was larger in the paediatric patients (−19% difference; *p* = 0.047) in age-stratified post hoc analysis [27]. Further, Zhu et al., also reported a statistically significant decrease in antibiotic prescriptions for discharged paediatric patients who underwent RRP testing in comparison to those not tested (8.8% versus 41.1%, *p* < 0.001) [35]. However, the utilisation of RRP testing was significantly less in the ED setting in comparison to inpatient wards (7.3% versus 78.9%, *p* < 0.001) [35]. Zhu et al., attributes this difference to the cost of RRP, however further research is required to assess the deterrents for effective ED use [35].

Although there is evidence for the use of RRP testing, there is limited evidence on the use of other diagnostic testing methods, such as chest radiographs and blood tests, as a method for antimicrobial stewardship. McDaniel et al., assessed adherence to diagnostic measures within an intervention including complete blood count, blood cultures, acute-phase reactants, and chest radiographs as an intervention to improve rates of appropriate antibiotic prescription and associated an increase in adherence (+10.8%, 95% CI 4.7%, 16.9%) with an increase in narrow-spectrum antibiotic prescription (8.3%, 95% CI 1.5%, 15.2%). Although there is a significant increase in both measures, there was no clear analysis conducted to ensure a correlation. Therefore, an RCT directly assessing the effect of diagnostic testing methods on antibiotic stewardship would be beneficial.

The success of an ASP cannot be completely appreciated without the clinical outcomes of the patients included in the study population. The majority of current literature focuses on the stewardship measures such antibiotic use and cost, however, more recently the literature is encouraging a shift in focus towards the clinical impact of ASPs [47]. Only two of our included studies reported patient clinical outcomes as a primary measure of their studies [25,28]. Van de Maat et al., reported ‘strategy failure’ as a primary outcome, defined as the need for further antibiotic prescription, rehospitalisation, persisting fevers or oxygen dependency within seven days or any other notable complications [28]. Similarly, Pernica et al., reported ‘clinical cure’ as their only outcome measure as they compared the success of a short course of antibiotics for community-acquired pneumonia in comparison to a long dose [25]. One included study reported patient outcomes as a secondary outcome referred to as ‘right care’, however did not report on specific patient outcomes following discharge from ED [30]. Further, four included studies addressed patient outcomes in their discussion, reporting that there were no significant differences in patient outcomes between the pre- and postintervention groups [13,27,31,33]. Five included studies did not to address patient outcomes throughout the study [24,26,29,34,35]. This highlights a gap in the current literature which needs to be addressed to ensure ASPs are being implemented safely as well as efficaciously. Therefore, future research on ASPs should always include clinical outcomes as a primary measure of success. Further, the distribution of ARTI emergency presentations across the paediatric population differs between paediatric age groups, with children aged <1 to 4 years old being the most frequent in comparison to children aged 5–18 years old [48]. Future research on paediatric ASPs should strive to define the most appropriate ASP design for different age groups within the paediatric population to ensure the applicability and effectiveness of intervention.

### Strengths and Limitations

This study has many advantages. The inclusion of only studies meeting the mandatory criteria and minimum score requirements from the ICROMS risk of bias tool, ensured only good-quality papers were reviewed. Further, a variety of ASP interventions were reviewed, providing an overview and opportunity for comparison. This provides a strong basis for future ASP development and implementation.

Our study has some limitations. Firstly, only four databases were searched, and some references may have been missed. Further, the search was restricted to papers published in English, which may have led to some relevant papers being overlooked. It should also be noted that with the high volume of observational studies included in this review, a risk of reporting bias is present however, this was minimised by the inclusion of only high-quality studies. Lastly, the phrase ‘antibiotic stewardship’, as a relatively new term, may not have applied to all studies exploring this concept which may have resulted in relevant papers being missed.

## 5. Conclusions and Recommendations

We conclude that most ASPs are effective in addressing suboptimal antibiotic prescription for respiratory infection in the paediatric emergency setting. Successful delivery of both education-based ASPs, as well as clinical tools such as RRP testing, translated to judicious antibiotic prescribing with a reduction in overall antibiotic prescribing and increased the proportion of narrow-spectrum antibiotics and short-course antibiotic therapies. As many included papers were not RCTs, there are many factors in the delivery of ASPs that further research can shed some light on. We recommend further research into the effectiveness of ASPs for respiratory infections in the paediatric ED setting. Such studies should aim to identify focused strategies to improve the adherence to and efficacy of ASPs and strive to include clinical outcomes as a primary measure of success.

## Figures and Tables

**Table 1:** Paediatric antimicrobial stewardship for respiratory infections in the emergency setting: a systematic review (1 August 2001 to 31 July 2021).

Author Year; Country; Study Period; Setting	Study Design; Population and Sample Size	Objective	Intervention	Key Findings
Ambroggio et al., 2013 [24]; USA; 1 May 2011–21 July 2012; Cincinnati Children’s Hospital Medical Centre (CCHMC)	Retrospective Cohort Study; 3 months–19 years, discharge diagnosis code of pneumonia (noncomplicated or pneumonia-related sx *n* = 217	Evaluate quality improvement in a setting without a formal ASP	- Guideline Seminar - One-page summary sheet outlining guidelines - Nurse Flag Cards - Index card with appropriate first line Abx information for physicians - Electronic medical record (EMR) changes to include hyperlink to guidelines and defaulted orders to appropriate Abx orders	Improvement in appropriate Abx prescribing in the ED following the guideline seminar (0% to 82%)
Forrest et al., 2020 [30]; USA; 90 days; Urgent Care Centre	Cohort Study; Adults and children with URIs and/or head, ears, nose, throat viral illnesses presenting to urgent care *n* = 279	Improve patient-centred right care for patients of 65 years and younger with URIs and/or head, ears, nose, throat viral illnesses presenting to ED from 36.2% to 80% within 90 days	Rapid-cycle Quality Improvement (QI) project with 4 × 2-weekly Plan-Do-Study-Act (PDSA) cycles: Team engagement: bi-weekly QI team meetings Patient engagement: shared decision aid Abx prescribing 5 DS tool Case Management Log	- Right care increased from a baseline of 36% to 78% during the study period. - Patient-centred engagement score rose from 33% to 93% - Right care proper Abx prescription increased from 63% to 91%
Huang et al., 2020 [32]; Taiwan; January 2008–December 2017; Taichung Veterans General Hospital	Retrospective noncontrolled before-and-after study; Three age groups (<3 years, 3–6 years, 7–18 years) Nasopharynx, throat swab, and sputum culture from children <18 years *n* = 914	Evaluate the impact of the implementation of the national PCY13 vaccination program and the 2013–2015 antimicrobial management project on antimicrobial drug susceptibility or respiratory tract bacteria in children	Three Temporal Stages: Pre-PCV13 vaccination era (2008–2012) Enhanced antibiotic control strategy era (2013–2015) - Project management centre, demonstration centre and three levels of hospital participation Post antibiotic control strategy era (2016–2017)	- Pneumococcal vaccine decreased infective pneumococcal disease in children and improved rates of antibiotic resistance in Taiwan. - Enhanced antibiotic control strategy improved drug resistance in nosocomial pathogens but had little effect on community-acquired pathogens.
May et al., 2019 [27]; USA; December 2016–April 2018, Over 2 winter seasons and 1 intervening non-respiratory season; Level 1 ED	Prospective Pilot RCT; >12 months old, had symptoms of URTI or influenza-like illness and not on Abx *n* = 191	Evaluate whether having rapid, multipathogen test results available during the ED visit would have a significant impact on management and outcomes in patients with clinical signs and symptoms of ARTI	Rapid, multipathogen respiratory panel (RP) test	Rapid RP testing associated with a trend towards decreased Abx use (–12% difference; *p* = 0.06/0.08, chi-square/Fisher exact test) that was larger in paediatric patients (−19% difference; *p* = 0.047/0.07) in an age-stratified post hoc analysis
McDaniel et al., 2018 [33]; USA; Preintervention: January–December 2015, Intervention: January–Feb 2016, Postintervention: March 2016–February 2017; Freestanding, tertiary children’s hospital	Noncontrolled before-and-after study; 2 months–18 y.o at ED with primary or secondary diagnosis of uncomplicated CAP. *n* = 544 (preintervention) *n* = 321 (postintervention) *n* = 290 (postintervention in freestanding hospital)	Examine whether implementation of a CAP pathway within 3 community hospital EDs and inpatient units improved process measures related to appropriate laboratory testing and antibiotic prescribing, and to compare performance on these measures between the community hospitals and a freestanding children’s hospital	Clinical decision tool (CDT) as a diagnostic aid for paediatric patients presenting with respiratory distress	Adherence to process measures increased postintervention for: appropriate lab testing, narrow-spectrum Abx stewardship and macrolide stewardship by 10.8% (95% CI = 4.7% to 16.9%), 8.3% (95% CI = 1.5% to 15.2%), and 3.1% (95% CI = −4.3% to 10.4%), respectively
Ouldali et al., 2017 [13]; France; November 2009–October 2014; 7 PEDS of Parisian university hospitals	Multicentric noncontrolled interrupted time series analysis; Paediatric patients visiting ED and diagnosed with ARTI. *n* = 242,534	Assess the impact of implementing the 2011 national guidelines on antibiotic prescriptions for ARTI in PEDs	Implementation of 2011 French guidelines through: - Scientific discussion among doctors - Education sessions for new physicians and residents biannually - Physician pocket guidelines - Feedback on performance during first year	- Antibiotic prescription rate per 1000 PED visits for ARTI was 51.0 preintervention (with a steady increase +0.1% per 15-day period) - Postintervention, there was a significant, immediate change in Abx prescription rate (−15.5%, *p* = 0.01) with a significant change in slope (-0.4% per 15-day period, *p* = 0.04) - Estimated cumulative effect of intervention by the end of the study on Abx prescription rate for ARTI per 1000 PED visits was -30.9% (95% CI, −42.5, −20.1) - Adjusted analysis gave the same results, with a cumulative effect of −28.4% at the end of the study - Abx prescription rate for viral ARTI for 1000 PED visits also significantly decreased (immediate effect: 40.8%; 95% CI, −79.1, −2.5; *p* = 0.03 and no change in slope)
Pernica et al., 2021 [25]; Canada; Data analysed 1 March–8 July 2020; EDs of McMaster Children’s Hospital and the Children’s Hospital of Eastern Ontario	Two-centre parallel group noninferiority RCT; 6 months–10 years having fever within 48 h, respiratory symptoms, chest radiography and a primary diagnosis of pneumonia. *n* = 281	Determine whether 5 days of high-dose amoxicillin for CAP was associated with noninferior rates of clinical cure compared with 10-days of high-dose amoxicillin	5 days of high-dose amoxicillin therapy followed by 5 days of placebo (intervention) vs. 5 days of high-dose amoxicillin followed by a different formulation of 5 days of high-dose amoxicillin (control)	- Clinical cure was observed in 101/114 children (88.6%) in the intervention group and in 99/109 (90.8%) in the control group in per protocol analysis (RD, −0.016; 97.5% CI, −0.087) - Clinical cure at 14–21 days was observed in 108/126 (85.7%) in the intervention group and in 106/126 (84.1%) in the control group in the intention-to-treat analysis (RD, 0.023; 97.5% CI, −0.061)
Rutman et al., 2017 [31]; USA; 1 August 2011–31 August 2013; Seattle Children’s Hospital, Tertiary, university-affiliated 350-bed freestanding	Retrospective cohort study; 2 months–18 years, assigned a primary ICD-9 diagnosis code associated with CAP	Determine the relationship between standardising ED and inpatient care for CAP and antimicrobial stewardship, clinical testing, and cost	CAP pathway implementation by the ED and inpatient pathway through multiple strategies: - Discussion at physician staff meetings - Email notifications - Mandatory web-based training - Copies of pathway outside patient rooms and in provider work areas	- No statistically significant differences between pre- and post-intervention groups - Small increase in the percentage of ED patients who received narrow-spectrum Abx with a shift from 57% to 67% after intervention - No significant change in ED chest radiography use, ED length of stay, % of CAP admissions or cost of care
Shishido et al., 2021 [26], Japan; April 2014–September 2019; Kobe Children’s Primary Emergency Medical Centre	Retrospective noncontrolled before-and-after study; Most common diagnosis upper RTI, followed by gastroenteritis and bronchitis; 129,156 and 28,834 patients in the pre- and postintervention periods	Evaluate the effects of a nudge-based ASP in reducing unnecessary third-generation cephalosporin (3GC) prescriptions in paediatric primary emergency care centre	The implemented ASP utilizes monthly newsletters that report current antimicrobial use patterns and prescribing targets	- Prescription numbers of 3GC and other antimicrobials decreased gradually over the study period (with some fluctuations indicative of seasonal variation) - The proportion of prescriptions for antimicrobial-unnecessary diseases decreased from 65.2% to 44.5% one year after intervention. - The number of prescriptions for antimicrobial-unnecessary diseases decreased by 67.2% after intervention
Van de Maat et al., 2020 [28]; The Netherlands; 1 January 2016–27 August 2017 (baseline period), 28 August 2017–12 March 2018 (rollout period), intervention phase every 4 weeks, data collected until 30 September 2018 when target sample size achieved; Eight EDs in the Netherlands	Stepped-wedge randomised trial; 1–60 months presenting with fever and cough or dyspnoea Control *n* = 572 Intervention *n* = 340	Safely reduce antibiotic prescription in children under 5 years with suspected lower RTI at the ED, by withholding antibiotics in children at low or intermediate risk of bacterial pneumonia, as predicted by the Feverkidstool	Antibiotics withheld in children with low or intermediate predicted risk of bacterial pneumonia, antibiotics prescribed in children with a high predicted risk (Validated clinical prediction model of Feverkidstool)	- Overall Abx prescription not reduced in the intervention phase (30% vs. 25%; [aOR] 1.07, 95% CI 0.57 to 2.01, *p* = 0.75) - Strategy failure decreased from 23% in the pre-intervention phase to 16% in the intervention phase (aOR 0.53, 95% CI 0.32 to 0.88, *p* = 0.01) - Per protocol analysis gave similar results as intention-to-treat analysis - Exploratory analyses intervention influenced risk groups differently (*p* < 0.01), resulting in a reduction in Abx prescriptions in low/intermediate risk group (17% to 6%; aOR 0.31 [95% CI 0.12 to 0.81, *p* = 0.02] - Nonsignificant increase in the high-risk group (47% to 59%; aOR 2.28 [95% CI 0.84 to 6.17, *p* = 0.09])
Weddle et al., 2017 [34]; USA; Chart review at 2 preintervention time points (3 m, 1 m before educational sessions) and 3 postintervention time points (1 m, 3 m, 9 m after educational sessions); 4 UCCs affiliated with a free-standing children’s hospital, UCC sites include both urban and suburban locations	Noncontrolled before-and-after study; Patients had one of these conditions: UTI, pharyngitis, SSTI, URI, AOM or ABS, most common diagnosis was URI, at 74% (2576/3496 patients) N = 26	To determine if educational sessions would reduce inappropriate antibiotic usage.	Members of the institution’s antimicrobial stewardship program team provided 30 min educational sessions for each of the selected diagnoses	- 2% reduction in inappropriate Abx prescribing (10% preintervention vs. 8% postintervention, *p* = 0.02) - Inappropriate antibiotic use decreased in those who attended the educational session (9% preintervention vs. 6% postintervention, *p* < 0.01) - No significant change in inappropriate Abx prescribing in providers not attending educational sessions (10% preintervention vs. 9% postintervention) - All diagnosis groups showed a decrease in inappropriate Abx prescribing except for SSTI and AOM - Wrong dosage observed in 22% (12/55) of patients with confirmed group A beta-haemolytic streptococcal pharyngitis - Patients’ demography impacted antibiotic prescribing - Self-pay patients were more likely to receive an inappropriate Abx for pharyngitis but were less likely to receive an Abx for diagnosis of SSTI - Old age correlated with likelihood of received Abx for viral URI diagnosis - Children older than 3 y were more likely to receive an inappropriate Abx - Children 1–3 y or older than 6 y were less likely to receive initial Abx than children outside these age ranges
Yadav et al., 2019 [29]; USA; July 2017–February 2018 at UC Davis and Harbor-UCLA, November 2017–February 2018 at CHCO, a 12-month baseline period for statistical analysis; five EDs and four UCCs	Pragmatic, cluster RCT, Licensed clinicians at the participating sites eligible, diagnoses (primary and secondary) from the ICD-10-CM codes consistent with antibiotic-nonresponsive ARI diagnoses	Compare the effectiveness of an antibiotic stewardship intervention adapted for acute ambulatory care settings to a stewardship intervention that additionally incorporates behavioural nudges in reducing inappropriate prescriptions.	Two interventions are compared: Adapted intervention that consisted of education for patients and providers using materials from CDC’s Get Smart (currently called Be Antibiotics Aware) campaign adapted for the acute care setting, led by a physician champion at each site. Enhanced intervention that incorporated the adapted Get Smart campaign, in addition to individualized audit and feedback, peer comparisons, and nudges.	- Antibiotic prescribing for ARI visits dropped from 6.2% (95% confidence interval [CI] = 4.5% to 7.9%) to 2.4% (95% CI = 1.3% to 3.4%) - A significant reduction in inappropriate prescribing after adjusting for health-system and provider-level effects from 2.2% (95% CI = 1.0% to 3.4%) to 1.5% (95% CI = 0.7% to 2.3%) with an odds ratio of 0.67 (95% CI = 0.54 to 0.82). - Difference-in-differences between the two interventions was not significantly different.
Zhu et al., 2019 [35]; USA; 16 December 2013–15 December, 2015; ProMedica Toledo Children’s Hospital	Retrospective Noncontrolled before-and-after study; 1 month−18 years with uncomplicated ARTI admitted into the hospital or ED (those in the ED, had to be discharged from the ED for inclusion) ED group: *n* = 939	Assess whether respiratory pathogen panel (RPP) testing decreases antibiotic days of therapy and length of hospital stay for paediatric patients with ARTI	Samples for RPP testing were collected via nasopharyngeal swabs. RPP was performed through PCR detection by BioFire FilmArray Assay which identifies common viral pathogens, as well as common bacterial pathogens	ED group: - No statistically significant difference in number of patients who received Abx on discharge from ED between the pre- and post-RPP study periods - In the ED population, patients testing positive with RPP received fewer discharge prescriptions for antibiotics than patients not tested (8.8% vs. 41.1%; *p* < 0.001). - RPP use was more prevalent in admitted patients than in ED patients (78.9% vs. 7.3%; *p* < 0.001)

**Table 2:** Outcome measures of interest reported in included studies.

		Outcomes				
Authors	Intervention	Reduction in Inappropriate Antibiotic Prescription	Reduction in prescription of Broad-Spectrum Antibiotics	Reduction in Duration of Antibiotic Therapy	Patient Clinical Outcomes	Reduction of AMR
Ambroggio et al. [24]	Multifaceted education-based intervention	ND	Appropriate first-line Abx prescription: 0% to 82%	ND	ND	ND
Forrest et al. [30]	Multifaceted education-based intervention	Appropriate Abx prescription increased from 63% to 91%	ND	ND	Increased from 36% to 78%	ND
Huang et al. [32]	Multifaceted education-based intervention	ND	ND	ND	ND	*p* < 0.05
May et al. [27]	Rapid, multipathogen respiratory panel test	−12%; 95% CI [−25% to 0.4%]; *p* = 0.06/0.08	ND	ND	ND	ND
McDaniel et al. [33]	Multifaceted education-based intervention	ND	−10.8%; 95%CI [−4.7% to −16.9%]; *p* < 0.001	ND	ND	ND
Ouldali et al. [13]	Multifaceted education-based intervention	−0.4% per 15-day period; *p* = 0.04	ND	ND	ND	ND
Pernica et al. [25]	Reduced antibiotic therapy duration	ND	ND	ND	RD, −0.016; 97.5% CI −0.087	ND
Rutman et al. [31]	Multifaceted education-based intervention	ND	−10%	ND	ND	ND
Shishido et al. [26]	Feedback, peer-comparison and nudge-based intervention	−67.2% Regression coefficient −0.58; *p* < 0.001	ND	ND	ND	ND
Van de Maat et al. [28]	Multifaceted education-based intervention	[aOR] 1.07; 95% CI 0.57 to 2.01; *p* = 0.75	ND	ND	[aOR] 0.53; 95% CI 0.32 to 0.88; *p* = 0.01	ND
Weddle et al. [34]	Multifaceted education-based intervention	−2% *p* = 0.02	ND	ND	ND	ND
Yadav et al. [29]	Feedback, peer-comparison and nudge-based intervention	OR = 0.67; 95% CI = 0.54 to 0.82	ND	ND	ND	ND
Zhu et al. [36]	Rapid respiratory pathogen testing	78.9% vs. 7.3%; *p* < 0.001	ND	ND	ND	ND

Green—reduction with statistical significance; Yellow—reduction withoyt statistical significance; ND—not done.

## Data Availability

All data is contained within this article.

## Appendix A

**Table A1:** Search Strategy Used for PubMed, MEDLINE, Embase and Cochrane Database of Systematic Reviews.

Step	Search Terms
1	‘antimicrobial stewardship’
2	‘antimicrobial control’
3	‘antibiotic control’
4	‘antibiotic stewardship’
5	‘child *’
6	‘paediatric’
7	‘pediatric’
8	‘infant’
9	‘neonat*’
10	‘respiratory tract infection’
11	‘chest infection’
12	‘lung infection’
13	‘pneumo *’
14	‘emergency’
15	‘emergency department’
16	‘acute care’
17	‘critical care’
18	‘urgent care’
19	1 OR 2 OR 3 OR 4
20	5 OR 6 OR 7 OR 8 OR 9
21	10 OR 11 OR 12 OR 13
22	14 OR 15 OR 16 OR 17 OR 18
23	19 AND 20 AND 21 AND 22

* wildcard in search term.

## Appendix B

**Table A2:** ICROMS Quality Criteria for Application per Study Design.

	Quality Criteria		Study Design **						
	**Dimension**	**Specific Criteria ***	**RCT**	**CBA**	**CITS**	**NCITS**	NCBA	CS	QUAL
1	Clear aims and justification	a. Clear statement of the aims of the research?	YY	YY	YY	YY	YY	YY	YY
		b. Rationale for number of pre- and postintervention points or adequate baseline measurement	N	N	Y	YY	YY	N	N
		c. Explanation for lack of control group	N	N	N	Y	Y	N	N
		d. Appropriateness of qualitative methodology	N	N	N	N	N	N	Y
		e. Appropriate study design	N	N	N	N	N	N	YY
2	Managing bias in sampling or between groups	a. Sequence generation	YY	N	N	N	N	N	N
		b. Allocation concealment	YY	N	N	N	N	N	N
		c. Justification for sample choice	N	N	N	YY	YY	N	N
		d. Intervention and control group selection designed to protect against systematic difference/selection bias	N	YY	N	N	N	N	N
		e. Comparability of groups	N	N	N	N	N	YY	N
		f. Sampling and recruitment	N	N	N	N	N	N	YY
3	Managing bias in outcome measurements and blinding	a. Blinding	YY	N	N	N	N	N	N
		b. Baseline measurement and protection against selection bias	N	YY	N	N	N	N	N
		c. Protection against contamination	N	YY	N	N	N	N	N
		d. Protection against secular changes	N	N	YY	N	N	N	N
		e. Protection against detection bias: Blinded assessment of primary outcome measures	Y	Y	Y	Y	Y	Y	N
		f. Reliable primary outcome measures	Y	Y	Y	Y	Y	Y	Y
		g. Comparability of outcomes	N	N	N	N	N	YY	N
4	Managing bias in follow-up	a. Follow-up of subjects (protection against exclusion bias)	Y	N	N	N	N	N	N
		b. Follow-up of patients or episodes of care	Y	N	N	N	N	N	N
		c. Incomplete outcome data addressed	Y	Y	Y	Y	Y	YY	Y
5	Managing bias in other study aspects	a. Protection against detection bias: Intervention unlikely to affect data collection	Y	Y	Y	Y	Y	N	N
		b. Protection against information bias	N	N	N	N	N	Y	N
		c. Data collection appropriate to address research aims	N	N	N	N	N	N	Y
		d. Attempts to mitigate effects of no control	N	N	N	YY	YY	N	N
6	Analytical rigour	a. Sufficient data points to enable reliable statistical inference	N	N	YY	N	N	N	N
		b. Shaping of intervention effect specified	N	N	Y	N	N	N	N
		c. Analysis sufficiently rigorous/free from bias	Y	Y	Y	Y	Y	Y	Y
7	Managing bias in reporting/ethical considerations	a. Free of selective outcome reporting	Y	Y	Y	Y	Y	Y	Y
		b. Limitations addressed	Y	Y	Y	Y	Y	Y	Y
		c. Conclusions clear and justified	Y	Y	Y	Y	Y	Y	Y
		d. Free of other bias	Y	Y	Y	Y	Y	Y	Y
		e. Ethics issues addressed	Y	Y	Y	Y	Y	Y	Y

* Quality criteria applicability to study designs: Y = criteria to be included in quality assessment for study design; YY = mandatory criteria to be met for quality assessment; N = criteria not to be applied in quality assessment for study design. ** Study designs: RCT = randomised controlled trial; CBA = controlled before-after; CITS = controlled interrupted time series; CS = cohort study; NCITS = noncontrolled interrupted time series; NCBA = noncontrolled before-after; QUAL = qualitative.

## Appendix C

**Table A3:** ICROMS Decision Matrix—Mandatory Criteria and Minimum Score of Study Type for Inclusion in Review.

Study Design *	Mandatory Criteria	Minimum Score **
RCT, cRCT	1a, 2a, 2b, 3a	22
CBA	1a, 2d, 3b, 3c	18
CITS	1a, 3d, 6a	18
NCITS	1a, 1b, 2c, 5d	22
NCBA	1a, 1b, 2c, 5d	22
Cohort	1a, 2e, 3g, 4c	18
Qualitative	1a, 1e, 2f	16

Studies must meet mandatory criteria and a minimum score to be included in review. * Study Designs: RCT = randomised controlled trial; CBA = controlled before-after; CITS = controlled interrupted time series; cRCT = cluster-randomized controlled trial; NCITS = noncontrolled interrupted time series; NCBA = noncontrolled before-after. ** Scores applicable to each criteria: Yes (criterion met) = 2 points; Unclear (unclear whether or not the criterion is met) = 1 point; No (criterion not met) = 0 points.

Appendix B and Appendix C adapted from Zingg et al., Innovative tools for quality assessment: integrated quality criteria for review of multiple study designs (ICROMS). Public Health 2016, 133, 19–37.

## Appendix D

**Table A4:** Score Attributed to Included Articles. Paediatric Antimicrobial Stewardship for Respiratory Infections in the Emergency Setting: A Systematic Review.

Study	Study Design	Minimum Score Required	Study Score
Ambroggio et al.	Cohort	18	28
Forrest et al.	Cohort	18	24
Huang et al.	NCBA	22	30
May et al.	RCT	22	35
McDaniel et al.	NCBA	22	34
Ouldali et al.	CITS	18	39
Pernica et al.	RCT	22	33
Rutman et al.	Cohort	18	32
Shishido et al.	NCBA	22	36
Van de Maat et al.	RCT	22	36
Weddle et al.	NCBA	22	26
